# Are all antibiotic persisters created equal?

**DOI:** 10.3389/fcimb.2022.933458

**Published:** 2022-08-17

**Authors:** Michael W. Shultis, Claire V. Mulholland, Michael Berney

**Affiliations:** Department of Microbiology and Immunology, Albert Einstein College of Medicine, New York, NY, United States

**Keywords:** mycobacteria, tuberculosis, multidrug, persister, persistence, tolerance, resistance

## Abstract

Antibiotic persisters are a sub-population of bacteria able to survive in the presence of bactericidal antibiotic despite the lack of heritable drug resistance mechanisms. This phenomenon exists across many bacterial species and is observed for many different antibiotics. Though these bacteria are often described as “multidrug persisters” very few experiments have been carried out to determine the homogeneity of a persister population to different drugs. Further, there is much debate in the field as to the origins of a persister cell. Is it formed spontaneously? Does it form in response to stress? These questions are particularly pressing in the field of *Mycobacterium* *tuberculosis*, where persisters may play a crucial role in the required length of treatment and the development of multidrug resistant organisms. Here we aim to interpret the known mechanisms of antibiotic persistence and how they may relate to improving treatments for *M. tuberculosis*, exposing the gaps in knowledge that prevent us from answering the question: Are all antibiotic persisters created equal?

## Introduction

In 2020 the WHO reported an estimated 10 million people contracted tuberculosis and 1.5 million died from the disease, making it the second most deadly infectious disease behind COVID-19 worldwide (WHO 2021). The agent responsible for this previously mysterious disease was first identified in 1882 when Robert Koch discovered the bacterium *Mycobacterium tuberculosis* as its cause (Murray et al., 2015). It wasn’t until the 1950s that reliable chemotherapy was developed (Murray et al., 2015). This began with streptomycin and para-aminosalycylic acid in 1944, but monotherapy treatments resulted in drug resistance, emphasizing the need for combination therapies. Isoniazid was introduced in 1951, leading to a combined “triple therapy”. This therapy required treatment times lasting up to 24 months long (Murray, 2004). Continued emergence of drug resistant populations led to the development of ethambutol in 1961 (Murray et al., 2015). It wasn’t until 5 years later that the introduction of rifampin combined with isoniazid was able to shorten treatment times to 9 months. Finally, the introduction of pyrazinamide further reduced treatment times to 6 months, bringing us to the combination therapies that remain in use today (Murray et al., 2015). The most common regimen includes the combination of isoniazid (INH), ethambutol (EMB), rifampicin (RIF), and pyrazinamide (PZA) for a period of 6 to 9 months (Nahid et al., 2016). Curiously, the two drugs that enabled greatly shortened treatment times, RIF and PZA, share a common property: the ability to kill persistent bacteria (Zhang et al., 2014; Hu et al., 2015).

### What is a persister?

**Figure 1:**
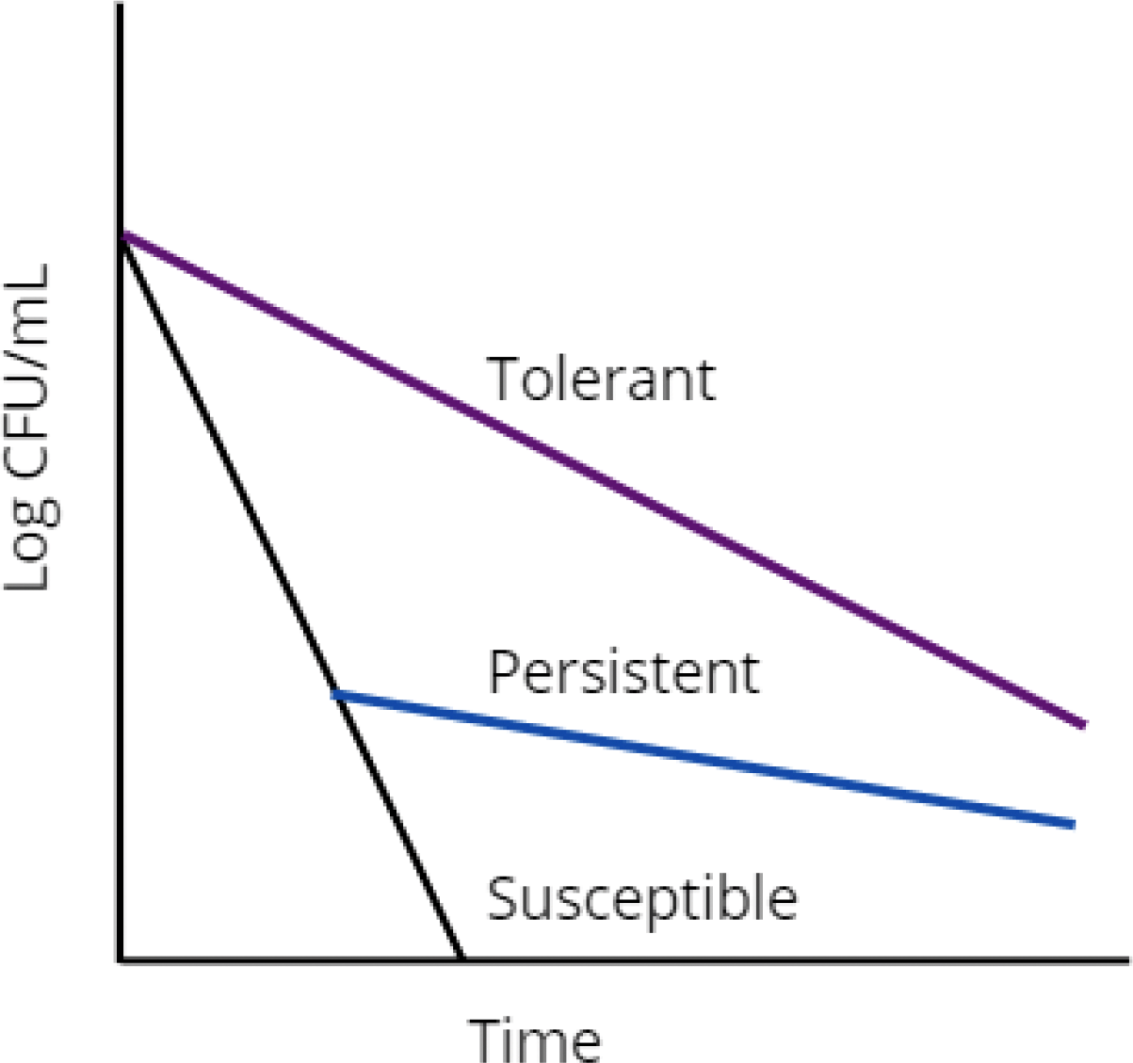
Graphical representation of drug susceptible (black), persistent (blue), and tolerant (purple) bacterial populations.

Persistent bacteria are a sub-population of bacteria that demonstrate slower killing kinetics in response to a stress, yielding a bimodal kill curve (**Figure 1**, blue line) (Boldrin et al., 2020). *Persistence* is distinct from antibiotic *resistance* because the state of persistence is non-heritable (Balaban et al., 2019). If persistent bacteria are regrown and exposed to the same stressor, they will again exhibit a heterogeneous response with a bimodal kill curve. Conversely, if a small subpopulation of resistant bacteria is isolated, regrown, and retreated with the same drug, growth would be observed instead of bimodal killing. Persistent bacteria exist across bacterial species, though are commonly referred to by the umbrella term ‘persisters’.

The first observation of antibiotic persistence was made in 1944 when Joseph Bigger demonstrated penicillin was incapable of sterilizing a culture of staphylococci (Bigger, 1944). In 1964 physicians with Cornell medical school made observations of “disappearing” *M. tuberculosis* bacilli in mice (McCune et al., 1966; McCune et al., 1966). Treatment of mice with a specific regimen of INH and PZA could push *M. tuberculosis* into an undetectable state by microscopy, culture, or reinfection. When treatment was removed for 90 days, *M. tuberculosis* became detectable in 1/3 mice (McCune et al., 1966). The undetectable state was only achievable when the mice were treated in order first with INH for a period of 2-4 weeks, followed by PZA for 8 weeks (McCune et al., 1966). If the duration of therapy was extended to 26 weeks no bacterial regrowth was observed up to 6 months after treatment cessation, suggesting these bacteria were persistent to sequential INH then PZA treatment after 12 weeks, but were killed with prolonged 26 week treatment (McCune et al., 1966). In these discussed conditions, treatment was generally carried out immediately following inoculation of mice. When mice were left untreated for 15 weeks, the undetectable state was not achievable with 12- or 26-week therapy. Among the bacilli that were detectable in the 26 week treated condition, only INH and PZA dual resistant mutant strains were identified, indicating that after 15 weeks enough pre-existing INH+PZA resistant mutants were generated, preventing the study of persistent bacteria (McCune et al., 1966). Although persistence is a common phenomenon in bacteria, a mechanism ubiquitously required for antibiotic persistence has yet to be identified, specifically in *M. tuberculosis*. The results described in the above Cornell study indicate that revealing the dynamics of persister cell formation are critical to accelerating the sterilization of *M. tuberculosis* infections.

Comprehensive and systematic reviews of persister phenomena have been presented elsewhere e.g (Boldrin et al., 2020). In the present review we focus on offering interpretations of the available data to illustrate the gaps in knowledge that prevent us from drawing conclusions about the heterogeneity of *M. tuberculosis* persisters to multidrug therapies.

### Tolerance versus persistence

The terms persistence and tolerance are often used interchangeably. Some prefer to draw a line between the two, describing them as similar but different phenomena (Brauner et al., 2016). The label of ‘persister’ appears largely to be definitionally confined. Under the current definition, an antibiotic persister must be a part of a small subpopulation, non-growing in the presence of the drug, genetically identical to the population that was killed by the stressor, vary only slightly with drug concentration at high concentrations of antibiotic, the list goes on (Balaban et al., 2019). By this definition, a bacterium could be labelled tolerant or persistent depending only on the characteristics of surrounding bacteria. If alone, this bacterium is a persister (**Figure 1**, blue line). If part of a larger population of bacteria exhibiting the same survival advantage, it is tolerant (**Figure 1**, purple line). There is no evidence to support the mechanistic distinction of persistent and tolerant bacteria, which is ultimately why many use these terms interchangeably. The mechanisms of *M. tuberculosis* tolerance are described in striking similarity to those of persistence. Metabolic slowdown, transcriptional and translational responses to stress, toxin-antitoxin system utilization, and efflux pumps are used to describe the mechanisms of both persistence and tolerance, [reviewed elsewhere in (Boldrin et al., 2020; Goossens et al., 2020)]. Comparatively it is very easy to draw the line between resistant bacteria and persistent bacteria. Resistance is a heritable adaptation that enables bacteria to grow in the presence of antibiotic (Balaban et al., 2019).

In summary, persistent bacteria are a drug tolerant sub-population that endures bactericidal antibiotic treatment through non-heritable mechanisms. Therefore, is it natural to wonder: If these survival advantages are non-heritable, where do persisters come from?

### The origins of persisters

The exact origins of persistent bacteria remain shrouded in mystery. Persisters have been described as belonging to two categories - type 1 and type 2 (Balaban et al., 2004). These types will be referred to here as triggered and stochastic persisters respectively. The distinction between these two categories can generally be understood as a description of when a persister is formed. Does a persister already exist in a population before a stress is introduced? Does a normally growing bacterium respond to stress in its environment by becoming a persister cell? Triggered persisters form in response to a trigger in their environment, whereas stochastic persisters form in the absence of external triggers (Balaban et al., 2004; Balaban et al., 2019). This distinction can be difficult to make when persisters make up 1% or less of a bacterial population (Lewis, 2007) and can only be definitively identified by their survival in response to a stress.

In *Escherichia coli* some studies have reported persistence arising in a stochastic pattern consistent with the decreased availability of nutrients that enhance ATP production in the cell (Shan et al., 2017; Manuse et al., 2021). This increase in persistence to ciprofloxacin and ampicillin is suggested to be due to decreased ATP generation, leading to lower activity of antibiotic targets, resulting in drug tolerance (Shan et al., 2017). This result was interrogated following single cells in solution, revealing 15 out of 16 ampicillin persister cells were not growing prior to ampicillin treatment, supporting their identification as stochastic persisters (Manuse et al., 2021). Other groups have identified *E. coli* persisters that originate from metabolically active cells (Dorr et al., 2010; Goormaghtigh and Van Melderen, 2019). In a single-cell experiment, microfluidics were used to image cells in a culture every 15 minutes, enabling retroactive observation of persister cell growth once they were identified by ofloxacin treatment (Goormaghtigh and Van Melderen, 2019). The authors tracked the cell area of persistent cells and were able to identify a rate of elongation immediately before ofloxacin treatment. Though a small significant decrease in growth rate was identified in persisters compared to the total population, the authors attributed this to a high statistical power as they were unable to find a significant difference when they randomly sampled a smaller subset of the non-persistent population (Goormaghtigh and Van Melderen, 2019). Another group found that ciprofloxacin, also a fluoroquinolone, was able to induce *E. coli* persister cell formation *via* the SOS response triggered by DNA damage (Dorr et al., 2010). In *Pseudomonas aeruginosa*, cells have been observed to upregulate persistence in response to quorum-sensing signals secreted into their media (Moker et al., 2010).

These points together indicate that the mechanisms for persister cell formation can vary between bacterial species and can vary depending on the stress applied to bacteria within the same species. When ampicillin was selected to reveal the *E. coli* persister population in the first experiment endorsing stochastic persistence, bacteria that may have been persistent to another drug were killed, preventing their characterization (Manuse et al., 2021). When ofloxacin or ciprofloxacin were used to reveal the *E. coli* persister population instead, a different group of bacteria capable of triggered persistence may have ended up being analyzed (Dorr et al., 2010; Goormaghtigh and Van Melderen, 2019). Both populations seem to exist, meaning the origins of persister populations can be varied. It is likely that stochastic persistence remains present at a certain level and that triggered persistence can occur with different intensities to different stressors.

Mycobacteria also demonstrate characteristics consistent both with stochastic and triggered persistence. The growth of mycobacteria are inherently heterogenous. The bacilli elongate asymmetrically on one of their two poles (Aldridge et al., 2012). The growing pole deemed the accelerator pole and the nongrowing pole deemed the alternator pole (Aldridge et al., 2012). During each division one daughter cell inherits the accelerator pole while the other inherits the alternator pole (Aldridge et al., 2012). Cell growth continues along the older of the two poles, requiring the alternator pole be converted to an accelerator pole (Aldridge et al., 2012). It was found that daughter cells inheriting the accelerator pole elongated faster than daughter cells inheriting the alternator pole (Aldridge et al., 2012). Further, in *Mycobacterium smegmatis*, it was found that accelerator cells were generally more susceptible to cell wall antibiotics like INH and alternator cells were generally more susceptible to RIF (Aldridge et al., 2012). This result is consistent with the observation that most antimycobacterial drugs have poor activity on slow growing cells, with RIF and PZA being the exceptions (Xie et al., 2005; Pullan et al., 2016). Deletion of *lamA*, the gene responsible for the inhibition of growth at new growth poles, results in a more symmetrical growth from each pole. Of note, these more uniform cells demonstrate a faster killing rate in response to RIF in *M. tuberculosis* (Rego et al., 2017). In *M. smegmatis* these cells were killed faster in response to RIF as well as cell wall targeting drugs (Rego et al., 2017). When considering this slow, asymmetric growth pattern of *M. tuberculosis* it isn’t difficult to rationalize that this heterogeneity could lead to stochastic persisters. Indeed, authors Jain et al. demonstrated using a dual reporter mycobacteriophage that genes linked to persistence in *M. tuberculosis* were upregulated prior to INH treatment, and that the bacteria expressing these genes were enriched in the persistent population (Jain et al., 2016).

In another vein, *M. tuberculosis* excels at responding to stressors in the host environment. Signal transduction systems have been shown to be essential for *M. tuberculosis* to establish latent infection in lung tissue and play a role in the response of *M. tuberculosis* to environmental stressors (Zahrt and Deretic, 2001; Bretl et al., 2011). Mistranslation has been shown to be more prevalent in stressed *M. tuberculosis*, which has led to increased bacterial survival to RIF (Javid et al., 2014). When sequenced, the surviving bacteria contained no mutations in the RIF resistance determining region (RRDR), suggesting these bacteria were demonstrating triggered persistence (Javid et al., 2014). Collectively these observations indicate that persistent *M. tuberculosis* is composed of a mixed population of pre-existing and triggered persistent bacteria. This heterogeneity is likely further exacerbated in the host environment where *M. tuberculosis* encounters a variety of stressors (Warner and Mizrahi, 2006; Adams et al., 2011; Liu et al., 2016).

### The nature of a persister

Regardless of its origin, it is important to consider the nature of a persister when attempting to design therapies to sterilize these bacteria. As discussed above, many drugs that impact the bacterial cell wall require actively growing cells to impact their targets (Xie et al., 2005). This fact has led to the belief that all persisters are non-growing cells, but is this truly the case?

Perhaps the most accepted characteristic of a persister is the characteristic of dormancy (Wood et al., 2013). Persisters are generally thought of as metabolically stunted bacteria that are inaccessible by antibiotics because the systems the antibiotics impact are inactive. It has been suggested that vitamin C and cysteine can prevent this metabolic shutdown by stimulating respiration, leading to sterilization of *M. tuberculosis in vitro* (Vilcheze et al., 2013; Vilcheze et al., 2017). In starved or stationary phase *E. coli* and *P. aeruginosa*, where bacteria metabolize more slowly, populations seem to be enriched for persistence (Keren et al., 2004a; Volzing and Brynildsen, 2015). This effect is observed in starved *M. tuberculosis*, where activation of the stringent response mediates persister formation, and deletion of a stringent response enzyme reduces persistence (Dutta et al., 2019). This stringent response enzyme, Rel, initiates metabolic arrest in *M. tuberculosis* (Dahl et al., 2003). The enrichment of persisters in stationary phase has been theorized to be a result of ATP-depletion (Manuse et al., 2021).

Contrarily, some groups presented evidence that persister cells can be metabolically active, and even actively dividing, in *E. coli* and *M. smegmatis* (Orman and Brynildsen, 2013; Wakamoto et al., 2013). In the case of *E. coli* 20/100 persisters were identified to be metabolically active by fluorescence-activated cell sorting (Orman and Brynildsen, 2013). This result comes with scrutiny as the experimental design is accused of carrying over persistent bacteria in the inoculum of the assay (Wood et al., 2013). *M. smegmatis* cells were observed to grow and divide in the presence of INH so long as expression of KatG, the activator of the prodrug INH, was suppressed (Wakamoto et al., 2013). Proponents of dormancy believe this to be an outlier, stating these cells are not persisters but instead normally growing cells that haven’t expressed KatG (Wood et al., 2013). This interaction is seen as unique because the cells aren’t under any stress from the antibiotic until converted to the active form, making this case inapplicable to persisters as a whole (Wood et al., 2013). Though model *M. smegmatis* is often used in experiments due to its faster growth and reduced biosafety requirements, it is important to be cautious when making direct comparisons to *M. tuberculosis* given the inherent differences of these two bacteria. However, we believe that when considering persistence in *M. tuberculosis* this result is important to consider given it is a direct study of mycobacterial persistence to a clinically relevant antimycobacterial drug. Furthermore, even *E. coli* persisters have been reported as having a reduced, but nonzero growth rate prior to antibiotic treatment (Balaban et al., 2004).

Briefly mentioned above, work in *M. smegmatis* has demonstrated bacterial growth in the presence of RIF, despite cells remaining genotypically sensitive to the drug (Javid et al., 2014). In this study, investigators generated a strain of *M. smegmatis* that resulted in higher rates of protein mistranslation, and these bacteria demonstrated 1000-fold more colonies on RIF containing agar, despite containing no mutations in the RRDR (Javid et al., 2014). The authors then utilized a bacterial strain with a higher fidelity ribosome, resulting in less mistranslation. This strain resulted in a decrease of bacterial survival to RIF compared to wild-type, suggesting that bacterial mistranslation is a unique way mycobacteria can survive antibiotic stress, though it is unknown if a resistance conferring mutation existed beyond the RRDR in the high protein mistranslation strain (Javid et al., 2014).

Another study was carried out in *M. smegmatis* and *M. tuberculosis* that demonstrated “semi-heritable” growth in the presence of RIF (Zhu et al., 2018). The cell wall of *M. smegmatis* was fluorescently labelled and then exposed to increasing concentrations of RIF. Cells that can grow will become less fluorescent as they “dilute” the fluorescent labels in their cell walls. It was found that unique to RIF, a small subset of cells was able to grow in the presence of RIF, up to 36 µg/mL (Zhu et al., 2018). The peak serum concentration for RIF appears to reach 3 to 5 µg/mL in humans (Seth et al., 1993; Lei et al., 2019). When *M. tuberculosis* or *M. smegmatis* exposed to RIF were plated on RIF-containing agar, a significant sub-population of colonies were observed (Zhu et al., 2018). When sequenced, all 30 of the present *M. smegmatis* colonies were found to be wild-type in the RRDR of the *rpoB* gene, the gene controlling the target of RIF. When these colonies were picked and re-plated onto RIF containing agar, there was a 10-fold increase in survival compared to the first exposure to the drug. When the same procedure was performed plating RIF-sensitive clinical isolates from active *M. tuberculosis* infections, a larger surviving sub-population was observed the longer the patient was on RIF therapy (Zhu et al., 2018). When these colonies were picked and regrown in non-selective media for 16 hours, no survival advantage was observed when compared to cells never exposed to RIF, making this effect “semi-heritable” (Zhu et al., 2018). This effect was correlated with increased transcription of *rpoB*.

Another study assessed the impact of asymmetric mycobacterial growth on RIF tolerance, and it was found that RIF tolerance was correlated with large cell size and older inherited growth poles (accelerator poles) (Richardson et al., 2016). This is consistent with the previously mentioned study demonstrating that alternator cells are more susceptible to RIF, since alternator cells will tend to be smaller on average, given the need to convert the nongrowing alternator pole to a growing pole (Aldridge et al., 2012). A study of persistent *M. tuberculosis* revealed persisters to PZA or RIF continue to engage in active transcription despite the apparent growth arrest associated with persistence (Hu et al., 2000). Given that mycobacteria seem to operate on a time based replication schedule, rather than a size based replication schedule (Aldridge et al., 2012), it becomes unclear if a persistent population is truly nongrowing or if this population is in a state of dynamic equilibrium between life and death, similar to the *M. smegmatis* cells observed by Wakamoto et al. (Aldridge et al., 2012; Wakamoto et al., 2013).

In this section we have discussed three instances of mycobacteria growing in the presence of antimycobacterial drugs, RIF and INH, despite remaining genotypically sensitive (Wakamoto et al., 2013; Javid et al., 2014; Zhu et al., 2018). These observations lend themselves to the conclusion that while most persisters are ‘dormant’, some persistent bacteria can grow in the presence of antimycobacterial drugs when treated with monotherapy.

### Phenotypic resistance

Now, with the arguments for non-dormant persister cells established, we introduce with this section the phenomenon of phenotypic resistance and discuss the roles this phenomenon may play alongside persistence and tolerance in complicating the treatment of *M. tuberculosis.*

As already discussed in the above section on tolerance and persistence, the line between tolerance and persistence is very thin and often crossed in discussions of both topics. In contrast, drug resistance has been clearly differentiated based on two factors, heritability and growth. However, discussion of metabolically active bacteria that survive antibiotic treatment begs the question: Are both parameters, heritability and growth, necessary to exclude the persister label or is heritability alone sufficient?

Phenotypic resistance describes a phenomenon where bacteria can grow in the presence of antibiotics, but the mechanism that enables their growth is non-heritable. This phenomenon was discussed above in the context of protein mistranslation (Javid et al., 2014), and is reviewed in greater depth elsewhere (Corona and Martinez, 2013). The bacteria mentioned previously did not contain mutations in the RRDR, indicating that if these bacteria were regrown and exposed to RIF under conditions that did not promote mistranslation, they would remain drug sensitive unless a resistance mutation was present outside the RRDR (Javid et al., 2014). To some the growth of these bacteria excludes the persister label, but why? As discussed previously, we highlight here three instances of mycobacteria that survive treatment with antimycobacterial drugs through a non-heritable mechanism (Wakamoto et al., 2013; Javid et al., 2014; Zhu et al., 2018). Each author used a different descriptor for their population of cells, dynamic persistence (Wakamoto et al., 2013), phenotypic resistance (Javid et al., 2014; Zhu et al., 2018), and tolerance (Zhu et al., 2018). These studies all focus on a sub-population of bacteria undergoing non-heritable (or semi-heritable), heterogenous mechanisms of survival in response to an applied antibiotic stress. If any of these mechanisms arise during an infection, they would likely prolong treatment times and warrant a solution. Though these mechanisms seem to be drug specific, there is evidence, expanded on in the next section, that patient noncompliance and drug pharmacokinetics can impact the effective concentrations of certain drugs in the lesions where *M. tuberculosis* is present (Kimerling et al., 1998; Tostmann et al., 2013; Prideaux et al., 2015). In these cases, bacteria exhibiting drug-specific persistence mechanisms that we would otherwise expect combination therapies to kill, may be of greater clinical concern.

The important take away from these experiments is the identification of the mechanisms that lead to bacterial survival. If these mechanisms, transcriptional downregulation and protein mistranslation, impacted more general pathways, the resulting bacteria may exhibit survival to a wider array of drugs, making them of even greater clinical concern. Therefore, it is important to consider these as persister mechanisms, rather than being concerned whether the resulting phenotype is labelled tolerant, phenotypically resistant, or ‘persistent’. These definitional limitations introduce obstacles in communication making it difficult to solve the underlying issue that motivates all this research, how do we improve treatment of *M. tuberculosis?*

### Persisters and drug resistance

As discussed above, *M. tuberculosis* is capable of adapting to changes to its environment, including entering states of metabolic inactivity, rendering most antimycobacterials ineffective until the bacteria reactivate (Connolly et al., 2007). Although, if indeed some bacteria exist in a state of ‘dynamic persistence’ (Wakamoto et al., 2013) or ‘phenotypic resistance’ (Javid et al., 2014; Zhu et al., 2018), there is some plausibility that these growing persistent populations may undergo spontaneous mutations that drive them towards resistance and out of persistence. Though there is little evidence demonstrating that resistant populations arise directly from persistent populations (Cohen et al., 2013; Sebastian et al., 2017), any bacteria that survive antibiotic stress can reactivate and grow (Hu et al., 2000; Wood et al., 2013; Boldrin et al., 2020). This cycle of reactivation in the event of improper treatment or poor adherence enables the rise of drug resistant populations (Zhang et al., 2012). This concern is amplified when considering the evidence that mycobacteria can develop phenotypic resistance due to errors in transcription or translation triggered by various stressors (Hu et al., 2000; Wakamoto et al., 2013; Zhu et al., 2018). Though these results were only demonstrated with monotherapy, the spectrum of drug-noncompliant patients is vast. Non-compliant patients range from those that take no drugs to those that miss some of their doses (Munro et al., 2007). In a study of *M. tuberculosis* infected patients in New York City in 1997, 48% were found to be nonadherent. Non-adherent patients took longer to recover and were more likely to develop drug resistant tuberculosis (Pablos-Mendez et al., 1997).

Even if patients remain adherent to therapy, there have been observations of patients with serum drug concentrations below the therapeutic range for these drugs (Kimerling et al., 1998; Tostmann et al., 2013). When drug concentrations fall below the level required to inhibit bacterial growth, drug resistant populations can arise. Furthermore, even when patients maintain therapeutic serum concentrations, authors Prideaux et al. demonstrated that different drugs INH, RIF, PZA, and moxifloxacin (MXF) have “different spatial and temporal patterns of distribution across TB lesion types and compartments” (Prideaux et al., 2015). Though it appears that INH demonstrated good penetration into critical compartments, INH never reached its minimum anaerobic cidal concentration (MAC), as the drug has poor activity on non-replicating bacteria. Inversely, MXF, a drug that has demonstrated promising activity against non-replicating bacteria *in vitro* but failed to shorten treatments in clinical trials (Li et al., 2015) demonstrated sub-cidal concentrations in regions of cavities containing non-replicating bacteria (Prideaux et al., 2015). The two drugs most active against persisters in this study, RIF and PZA, achieved cidal concentrations within relevant compartments of the studied lesions (Prideaux et al., 2015). These results indicate that bacteria exhibiting drug-specific persistence mechanisms may still contribute to the rise of drug resistant bacteria in compartments of relative monotherapy. Of particular concern are persisters to RIF and PZA or the persisters generated to second line therapies used to treat RIF and PZA drug resistant strains, discussed later this section.

The development of drug resistant *M. tuberculosis* can be considered a stepwise process (Allue-Guardia et al., 2021). Before acquiring “high-level” resistance mutations typically associated with clinical strains of *M. tuberculosis*, strains may first accrue “low-level” resistance mutations that enable the bacteria to survive higher concentrations of antibiotics before undergoing cell death (Safi et al., 2013). The importance of low-level resistance to the pathogenesis of *M. tuberculosis*, has been discussed well in a review on the evolution of antibiotic resistance in *M. tuberculosis* (Fonseca et al., 2015). Scientists studying the development of EMB resistance in clinical isolates suggest that these low-level resistant mutants are preferentially selected in patients exposed to sub-therapeutic drug concentrations (Safi et al., 2013). These low-level resistance mutations are typically thought to be associated with efflux pump systems (Machado et al., 2012). As the strains accumulate low-level resistance mutations, they provide a background for high-level resistant mutants to arise from (Martins et al., 2009). Aside from the upregulation of efflux pumps, other mutations that increase antibiotic tolerance have been implicated in the development of high-level drug resistant *M. tuberculosis* (Allue-Guardia et al., 2021). Two such mutations include transcription factor *prpR* and the gene encoding glycerol-3-kinase, *glpK*, which have been demonstrated to promote drug tolerance to clinically relevant antimycobacterial drugs (Hicks et al., 2018; Bellerose et al., 2019). These examples demonstrate the role that persistence could be playing in the development of drug resistant *M. tuberculosis.*

Among persister populations may exist low-level resistant mutations that would otherwise not survive if not for the protection offered by persistence. As time goes on, in some patient’s compliance decreases (Jin et al., 2008), in others their metabolism induces sub-therapeutic drug levels (Kimerling et al., 1998), or in some lesions drug concentrations poorly penetrate compartments of lesions (Prideaux et al., 2015) providing the opportunity for either dynamic persistence (Wakamoto et al., 2013; Javid et al., 2014; Zhu et al., 2018) or low-level resistance mutations to exert their survival benefit. Subsequent growth leads to accumulation of low-level resistance or drug tolerance mutations until eventually strains are fully drug resistant. Once fully drug resistant, strains begin to undergo compensatory mutations to reduce the fitness cost of preliminary resistance mutations, resulting in clinically relevant drug resistant strains (Comas et al., 2012). The concept of compensatory mutations is reviewed well elsewhere (Castro et al., 2020). The bacterial mechanisms discussed in previous sections result in greater bacterial survival to monotherapy and therefore increased risk of spontaneous drug resistance, further underscoring the need for multidrug therapy when treating *M. tuberculosis*.

Once drug resistant organisms develop, the most concerning strains are those resistant to RIF and PZA. So long as strains are susceptible to these two drugs, treatment times remain between 6-9 months (Mase and Chorba, 2019). PZA resistant but RIF susceptible strains require 9-month treatment, RIF resistant but PZA susceptible strains require 12–18-month treatment, and strains resistant to both of these drugs require 18-month treatment, the same time required prior to the discovery of these two drugs (Mase and Chorba, 2019). Development of new drugs for *M. tuberculosis* has been slow, with only 3 new drugs, pretomanid, delamanid, and bedaquiline being approved in the last 40 years (Murray et al., 2015). Two of these drugs, bedaquiline and delamanid, have demonstrated activity against dormant bacteria (Koul et al., 2008; Chen et al., 2017). These drugs are typically reserved for multidrug resistant cases, but as evidenced by the treatment times of multidrug resistant bacteria, neither of these have demonstrated the same impact on treatment times as RIF and PZA (Mase and Chorba, 2019).

While we are unaware of studies comparing treatment outcomes of INH monoresistant (INHR) *M. tuberculosis* directly to RIF monoresistant (RIFR) *M. tuberculosis* in the same patient populations, studies of these monoresistant strains have been carried out in separate patient populations. In the following paragraph we discuss the general trend that patient outcomes for RIFR *M. tuberculosis* are worse than those of INHR *M. tuberculosis*. Given the knowledge that RIF has greater activity on persistent *M. tuberculosis* than INH, we suggest that the reason for these worse outcomes can be attributed to the reduced killing of persistent bacteria that RIF otherwise provides.

In 2019 a meta-analysis found the success rate for drug susceptible *M. tuberculosis* infections was 80.1%, multidrug resistant *M. tuberculosis* was 58.4%, and extensively drug resistant *M. tuberculosis* was 27.1% (Chaves Torres et al., 2019). Success was defined as patients that fit the criteria for “cure” or “treatment completion”. In one retrospective cohort analysis RIF monoresistance was found to occur less than INH monoresistance with 178 cases compared to 3469 (Prach et al., 2013). In this study it was concluded that compared to drug susceptible strains, patients with RIF resistant *M. tuberculosis* were twice as likely to die (Prach et al., 2013). Another study of 39 patients with RIF resistant *M. tuberculosis* identified only 20 patients that were cured. Of the 39 patients only 30 could be assessed for outcome as the other 9 had either died or been lost to follow up (Meyssonnier et al., 2014). In a study of 165 patients with INH resistant *M. tuberculosis* 140 had treatment success, while 12 had an unsuccessful outcome (Romanowski et al., 2017). The issue of RIF monoresistance was reviewed well by Malenfant and Brewer in 2021 (Malenfant and Brewer, 2021).

Given the heterogeneity of patient populations across these studies, it is difficult to draw conclusions about the outcomes of RIF monoresistant *M. tuberculosis* compared directly to other monoresistant strains. However, from the studies presented here it appears that patients with INHR *M. tuberculosis* experience more positive outcomes than those with RIFR *M. tuberculosis*. If further analyses were carried out that validated this trend that RIF resistance leads to worse outcomes than other resistances, this could illustrate further the importance of persisters and RIF’s role in killing them to patient outcomes. Persistent bacteria that would otherwise be killed by RIF may serve as a reservoir for the rise of multidrug resistant strains in compliant patients, as discussed above. In lieu of this line of experimentation, it is evident that rifampicin resistant strains require the longest treatment times (Mase and Chorba, 2019). It has been documented that longer treatment regimens have a lower compliance rate than shorter treatment regimens, and as discussed low adherence can lead to drug resistant populations (Jin et al., 2008).

### Multidrug persistence

It is common for persisters to be deemed or implied to be ‘multidrug tolerant’ (Keren et al., 2004b; Willenborg et al., 2014). In this context, multidrug tolerance means that when bacteria are grown, split into different aliquots, and treated with a range of different drugs, each aliquot demonstrates a persister population. Though persister populations are more and more frequently referred to as heterogenous, descriptions such as these paint a less clear picture. In two such studies a range of drugs were administered to *Streptococcus suis* and *E. coli* (Keren et al., 2004b; Willenborg et al., 2014). Analysis of kill curves performed in these two studies reveal persister populations that vary depending on the drug used in the experiment. As discussed earlier in this review, these populations are likely a mixture of pre-formed stochastic persisters and triggered persisters formed in response to the applied antibiotic stress. However, since this is still up for debate its informative to analyze these results under both paradigms.

Going stepwise, the first analysis comes through the lens of stochastic persistence. Since these bacteria are obtained from the same culture prior to antibiotic treatment, it is safe to assume the level of persistent bacteria should be the same prior to treatment. In the case of the *E. coli* experiment (Keren et al., 2004b), the clearest difference is between ofloxacin, a DNA gyrase inhibitor, and tobramycin, a bacterial ribosome inhibitor. Treatment with ofloxacin revealed a persister population approximately 2-3x in size to the tobramycin treated population (Keren et al., 2004b). Assuming pre-existing persister populations of the same size, this indicates that the true persister population size is closer to that revealed by ofloxacin and that the population observed after tobramycin treatment is a smaller portion of that larger population. This begs the question, is something about these bacteria different? Why did these bacteria persist to this point in the presence of ofloxacin, but not in the presence of tobramycin? This could be attributed to the efficacy of the drug. For example, RIF has a greater capacity to kill *M. tuberculosis* persister cells, but has very little impact on persister cells of *S. suis* or *Borrelia burgdorferi* (Keren et al., 2004b; Hu et al., 2015; Feng et al., 2015).

**Figure 2:**
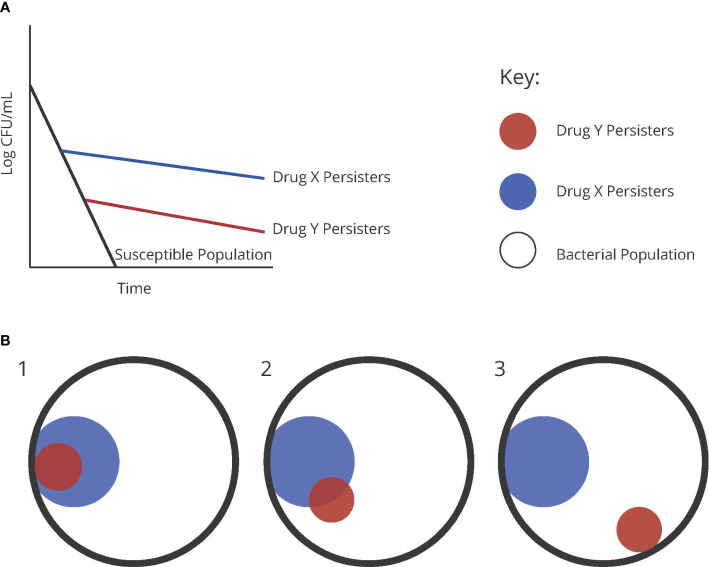
**(A)** Graphical representation of persister populations to two theoretical drugs, x and y, compared to a susceptible population of bacteria. **(B)** Diagrams representing the possible distributions of heterogenous persister populations of two different antibiotics.

To illustrate the concepts discussed here, we generated the schematic seen in **Figure 2**. This schematic illustrates a susceptible population compared to persistent populations observed to two different drugs, x and y (**Figure 2A**). In this case, assuming the persistent bacteria are the same or persistent *via* the same mechanism, tobramycin may be demonstrating a higher efficiency in killing these persistent bacteria than ofloxacin (**Figure 2B1**). The alternative explanation is that these bacteria are different, and that the populations revealed are engaging in two distinct mechanisms that confer persistence to one drug or the other (**Figure 2B3**), possibly with some overlap (**Figure 2B2**).

Through another lens, this higher amount of persisters in the ofloxacin group could be a result of triggered persistence, since ofloxacin and the similar drug ciprofloxacin have both been shown to induce persistence in metabolically active cells (Dorr et al., 2010; Goormaghtigh and Van Melderen, 2019). This explanation continues to beg the question whether triggered persisters are the same as the pre-existing persisters. The only way to discern this concept of cross-persistence is to sequentially treat a population with one drug and then the other, assessing what is commonly referred to as ‘cross-tolerance’.

This line of experimentation was carried out on *E. coli* with the drugs ciprofloxacin, ampicillin, rifampin, streptomycin, tetracycline, and levofloxacin (Wiuff et al., 2005; Singh et al., 2010). The results revealed that while some drugs conferred cross tolerance, this cross tolerance was not observed for all drugs even among those that did exhibit cross tolerance in certain combinations. For example, streptomycin persisters demonstrated cross tolerance to ampicillin, but not ciprofloxacin. Ciprofloxacin persisters demonstrated cross tolerance to RIF, but not streptomycin. Rifampin persisters did not exhibit cross tolerance to any drugs (Wiuff et al., 2005). However, even when cross-tolerance was not observed, sterilization did not occur, indicating possible sub-populations within persister populations that were cross-tolerant (Wiuff et al., 2005). Another experiment showed that *E. coli* persisters to streptomycin, ampicillin, and levofloxacin seem to all exhibit cross tolerance (Singh et al., 2010). These results suggest that persisters to one drug are not the same as persisters to another drug and may arrive in the persister state *via* unique mechanisms. In fact, RelE homologues, a toxin module found in *E. coli* and *M. tuberculosis* have demonstrated differential impacts on persistence to different antimycobacterial drugs in *M. tuberculosis* (Singh et al., 2010).

Toxin-antitoxin systems are used by many bacterial species. In these systems, bacteria synthesize both a toxin capable of suppressing cellular processes, as well as an anti-toxin capable of binding the toxin and preventing its effects (Boldrin et al., 2020). The anti-toxin tends to degrade much more rapidly than the toxin, requiring active upkeep to maintain antitoxin levels. DNA toxins can impact DNA gyrase, polymerase, or even cleave DNA directly (Bernard and Couturier, 1992; Critchlow et al., 1997). RNA toxins can degrade tRNAs and mRNAs or impair their activity through chemical modifications such as acetylation and phosphorylation (Mets et al., 2017; Culviner and Laub, 2018; Mets et al., 2019). These systems are covered well in a review on toxin-antitoxin systems (Jurėnas et al., 2022).

RelE is an mRNA toxin that cleaves mRNA entering the ribosomal translation site (Pedersen et al., 2003). In the case of RelE homologues in *M. tuberculosis*, RelE2 overexpression increased bacterial persistence to RIF, while its deletion decreased persistence to RIF. RelE3 overexpression increased persistence to INH, while its deletion decreased persistence to INH, but not EMB (Singh et al., 2010). When the authors assessed *M. tuberculosis* for cross-tolerance they found no cross-tolerance between INH, RIF, and PZA (Singh et al., 2010).

Other investigators Grant et al. observed persister populations in *M. smegmatis* and *M. tuberculosis* to drug combinations INH+RIF, Ciprofloxacin (CIP) +RIF, and OFX+INH, suggesting the presence of cross-persistence to these combinations. Interestingly, the persister population generated by 7 days of treatment with a combination of CIP and INH demonstrated cross-tolerance to RIF (Grant et al., 2012). The authors found accelerated killing of these persistent populations when cultures were maintained at a high level of dissolved oxygen (Grant et al., 2012). Though this condition would be difficult to maintain in hypoxic granulomas, this result indicates that an underlying process can be activated by the presence of oxygen in these cross-tolerant populations, either awakening them from the persister state or promoting death of persistent bacteria.

Though most of these studies of persistence have been carried out *in vitro*, experiments performed by Bellerose et al. have shown signs of multidrug-persistence *in vivo* (Bellerose et al., 2019). These authors inoculated mice with wild-type *M. tuberculosis* as well as a *glpK* mutant. As mentioned in the previous section, this *glpK* mutant is unable to phosphorylate glycerol, making the mutant incapable of utilizing glycerol metabolism in the host. The authors demonstrated that this mutant does not exhibit a growth disadvantage in the lungs of mice up to 40 days post infection but when exposed to antimycobacterial PZA, *glpK* mutants in the lungs demonstrated a significant survival advantage compared to the wild-type. Further, when mice were infected with *M. tuberculosis* and treated with INH, EMB, RIF, PZA, the authors identified varying amounts of surviving bacteria. While these surviving bacteria could be interpreted to be persistent, it is difficult to make that claim given bacterial counts were only obtained at a single time point after treatment, so no biphasic killing can be observed (Bellerose et al., 2019). More interestingly, when all four drugs were given in combination, the amount of surviving bacteria most closely resembled the amount surviving after PZA monotherapy (Bellerose et al., 2019). This may be indicative that the mechanism of persistence to PZA that *glpK* mutants undergo *in vivo* demonstrate cross-persistence to other drugs in the standard *M. tuberculosis* drug regimen. One limitation to this interpretation is the fact that drugs were administered as a combination therapy, making the potential mechanisms of persister cell formation much more complex than if drugs were administered in sequence (Bellerose et al., 2019).

It is logical to think that drug-specific INH persisters that are actively growing so long as they downregulate KatG would remain susceptible to other antimycobacterials (Wakamoto et al., 2013) and that those with phenotypic resistance to RIF due to alterations in *rpoB* transcription would remain susceptible to other antimycobacterials (Zhu et al., 2018). It is unclear from any of these studies if the persisters generated will persist when exposed to other antimycobacterials with different mechanisms. Though some investigations begin to show avenues for killing multidrug persistent *M. tuberculosis*, an additional universal mechanism that is more general to all antibiotics may be occurring in the background of these experiments. It would be very illuminating to identify the presence of one of these general mechanisms, as they may lie at the heart of improving treatment of *M. tuberculosis* infections.

### Why does it matter?

So far, we discussed that persistent bacteria are relevant for their potential role in *M. tuberculosis* infection severity, treatment times, and development of drug resistant populations. With this importance in mind, we set out to establish the origin, nature, and heterogeneity of persistent *M. tuberculosis*.

The origin of persisters is important to consider in the context of an infected patient. Which persisters are already present in a patient because of the various host environments *M. tuberculosis* finds itself in (Adams et al., 2011; Liu et al., 2016)? Which persisters do we induce when we give treatment? Answering these questions should shape how we configure and administer drug combinations.

The nature of persistent bacteria is important to consider in the evolution of drug resistance. History has already taught us the lesson that *M. tuberculosis* requires a multidrug chemotherapy and never to add a single drug to a failing regimen. Evidence discussed here reveals that dynamic persistence may play a role in this process of single drug resistance.

The heterogeneity of persistent *M. tuberculosis* is important to consider in improving treatment of both drug susceptible and drug resistant *M. tuberculosis*. Given the lack of study on cross-tolerance, it is unclear whether dynamic persistence exists to the multidrug regimens administered to patients. Patient non-compliance aside, it is also of concern whether drugs remain within the therapeutic range for all compliant patients (Kimerling et al., 1998; Pasipanodya et al., 2012; Tostmann et al., 2013). Drug concentrations outside the therapeutic range may enable dynamic persistence or regrowth despite the administration of combination therapy. The possibilities are endless, but the data are shallow. If persister populations vary in the presence of different drugs as argued here, then what are the next steps?

As illustrated in numerous examples discussed in this review, RIF appears to be key in the killing of persistent bacteria, with pyrazinamide a few steps behind. In the short term, we suggest that it is imperative to find new compounds that replicate RIF’s success in killing persistent bacteria in order to shorten treatment times and improve outcomes for RIF resistant *M. tuberculosis*. Salvage of efficacious antibiotics has previously been achieved using adjuvants such as beta-lactamase inhibitors or drug-drug conjugates such as tobramycin-ciprofloxacin (Maiti et al., 1998; Gorityala et al., 2016). One such attempt at a drug-drug conjugate was attempted for RIF by linking it to clofazimine (Saravanan et al., 2021). Though this conjugation did not result in a compound effective against RIF resistant *M. tuberculosis*, it did show activity at lower concentrations than either of the individual compounds. Whether through an adjuvant, RIF analogues that can impact clinical resister mutations, or new drugs that can kill the same persistent bacteria that RIF can, avenues like these could provide a short-term improvement to RIF resistant *M. tuberculosis*.

In the longer term it is imperative to identify general mechanisms of persistence in *M. tuberculosis*, particularly those mechanisms of persistence present in RIF persisters. Any new treatment capable of killing RIF persisters may hold promise to shorten the current treatment of drug susceptible *M. tuberculosis* and provide alternatives in the case of RIF resistance. Before these treatments can be developed, mechanisms of persistence must be further identified and currently known mechanisms of persister enrichment need to be screened for multidrug tolerance to ensure redundancy is avoided.

### Conclusions: Are all antibiotic persisters created equal?

Finally, we return to our initial question. Current knowledge on this topic paints a very multifaceted picture of persistence. Convincing data exist to support the existence of some general mechanism of persistence that leads to a basal level of stochastic persisters in a population (Shan et al., 2017; Manuse et al., 2021). Convincing data exist that support the process of triggered persistence, suggesting a more adaptive mechanism to arrive in the persister state (Dorr et al., 2010; Goormaghtigh and Van Melderen, 2019). As argued here, some experiments suggest that persistent bacteria engage in different mechanisms of persistence to survive the presence of different drugs (Singh et al., 2010). Of the data that exist, it appears persistent bacteria to one drug do not necessarily persist in the presence of another (Wiuff et al., 2005; Singh et al., 2010). To our knowledge, neither data exist to suggest stochastic and triggered persisters are composed of the same subpopulation of bacteria, nor does data exist to suggest INH and RIF persisters are composed of the same subpopulation of bacteria. Therefore, based on the current information available, we must assert that no, not all antibiotic persisters are created equal.

## Author contributions

MWS, CVM, and MB conceptualized and designed the review. MS wrote the first draft of the manuscript. All authors contributed to manuscript revision, read, and approved the submitted version.

## Funding

This work was supported by Potts Memorial Foundation and NIH grants T32AI007501 (supporting MWS) and R01AI139465 (supporting CVM and MB).

## Conflict of interest

The authors declare that the research was conducted in the absence of any commercial or financial relationships that could be construed as a potential conflict of interest.

## Publisher’s note

All claims expressed in this article are solely those of the authors and do not necessarily represent those of their affiliated organizations, or those of the publisher, the editors and the reviewers. Any product that may be evaluated in this article, or claim that may be made by its manufacturer, is not guaranteed or endorsed by the publisher.
